# A Conceptual Nutrition Literacy Framework for Adults in the United States: A Scoping Review and Thematic Analysis

**DOI:** 10.1016/j.advnut.2026.100686

**Published:** 2026-06-18

**Authors:** Aubree L Hawley, Sydney Boudrey, Erin K Howie, Xinya Liang, Samuel Selorm Attu, Keith S Santos, Jamie I Baum

**Affiliations:** 1 Center for Human Nutrition, University of Arkansas System Division of Agriculture, Fayetteville, AR, United States; 2 Department of Food Science, Dale Bumpers College of Agricultural, Food and Life Sciences, University of Arkansas, Fayetteville, AR, United States; 3 Department of Health, Human Performance and Recreation, College of Education and Health Professions, University of Arkansas, Fayetteville, AR, United States; 4 Rehab, Human Resources, & Communication Disorders, College of Education and Health Professions, University of Arkansas, Fayetteville, AR, United States

**Keywords:** nutrition literacy, health literacy, framework, concept mapping, functional literacy, interactive literacy, critical literacy

## Abstract

Poor dietary behavior is a major contributor to preventable chronic conditions in the United States. Although nutrition literacy (NL) is increasingly recognized as an important determinant of diet quality, the lack of a unified framework has limited its use in education, assessment, and intervention design. Therefore, this scoping review and thematic analysis aimed to synthesize existing NL definitions, frameworks, and measures to develop an evidence-based conceptual framework to guide assessment and intervention efforts among United States adults. A literature search was conducted using 4 databases: PubMed, Web of Science, CINAHL, and Google Scholar (inception to August 2025). Peer-reviewed studies were included if they examined NL frameworks, models, programs, interventions, assessment tools, or reviews. Studies were excluded if they focused on minors (<18 y of age), combined constructs of nutrition and food literacy, or are observational studies without tool/intervention development. Two independent reviewers screened and extracted study data in Covidence, resulting in 43 included studies. Reviewers then analyzed textual content to create themes using Nutbeam’s functional, interactive, and critical literacy domains. Thematic analysis allowed for concept mapping of emerging themes. Experts (*n* = 5) reviewed the preliminary framework using a structured 25-item internal questionnaire. Quantitative and qualitative feedback informed refinement of domains and framework organization. A total of 105 unique NL themes were identified and organized into 11 levels: knowledge, understand, obtain, apply (functional), advanced cognitive and application, motivation, communication (interactive), appraisal, advocacy, and translational (critical). The resulting NL conceptual framework defines NL as a multidimensional continuum encompassing functional, interactive, and critical domains for accessing, understanding, communicating, and applying nutrition information. This framework provides a foundation to support consistent measurement and inform the development of NL assessments and interventions for United States adults.

**Statement of significance**

This work establishes the first evidence-based comprehensive framework of nutrition literacy for United States adults, providing a unified structure to inform measurement and intervention design.

## Introduction

Preventable chronic conditions such as cardiovascular disease, cancer, stroke, and type 2 diabetes are major causes of death and disability in the United States [1]. Poor diet quality is a key modifiable contributor to these conditions, yet only 1.6% of United States adults meet criteria for ideal diet quality based on the American Heart Association 2020 continuous diet score with higher scores based on higher intake of fruits, vegetables, whole grains, fish, shellfish, nuts, seeds, and legumes and lower intake of sugar-sweetened beverages, processed meat, saturated fat, and sodium [2]. Furthermore, diets high in ultraprocessed foods, calories and sodium, and low in fruits and vegetables are especially common among individuals with limited health literacy [3,4] and nutrition literacy (NL) [5].

Health literacy refers to the ability to access, understand, and use health information. Greater health literacy supports informed decision making and navigation of complex health systems [6] and is associated with improved health outcomes and lower diet-related chronic disease mortality [7]. NL, a subdomain of health literacy, is positively associated with diet quality [8] and quality of life [9]. It encompasses not only nutrition knowledge but also the ability to apply information and critically evaluate dietary choices [10]. NL is defined inconsistently throughout the literature, with approximately 8 original definitions primarily focusing on a singular literacy component (e.g. functional literacy) [5,11]. In 2018, a systematic review was published identifying that the NL concept has been broadened to encompass the ability to acquire, understand, evaluate, and apply nutrition information to make appropriate nutrition decisions that promote health [12].

In 2015, Velardo [10] proposed Nutbeam’s tripartite model of health literacy [13,14] to define and advance the field of NL. Velardo [10] described NL as a multidimensional construct embedded within Nutbeam’s health literacy framework, encompassing functional (knowledge, understand, obtain, and apply), interactive (advanced application), and critical (appraisal and social action) domains. Building on this foundation, subsequent international NL models or assessment tools have adopted Nutbeam’s tripartite structure [12], while more recent work has expanded its scope to include factors such as structural barriers, systemic inequities, digital health literacy, and motivation in shaping nutrition-related behaviors [15,16]. Despite these advancements, definitions, conceptual models, and assessment tools are lacking or remain inconsistent, limiting the ability to measure NL effectively and compare outcomes across the literature [12].

The absence of a standardized NL conceptual framework, specifically in the United States, has resulted in fragmented approaches to defining and measuring NL across studies [5]. Existing tools vary widely in scope, focus, and theoretical grounding as some emphasize factual nutrition knowledge [8,[17], [18], [19]], while others assess skills in communication [20] or critical evaluation [21]. In contrast, other countries [11,21,22], particularly China [9,15,[23], [24], [25]], have made notable progress in developing and validating population-specific NL frameworks and assessment tools. Despite international advancements, United States efforts remain inconsistent, and a framework is yet to be established.

Without a unified framework, it also remains unclear how NL develops across domains or how it should be systematically targeted through education and public health interventions [12]. To address this gap, we conducted a scoping review and concept analysis to synthesize existing concepts, frameworks, and measures of NL among adults. The resulting framework was informed by Nutbeam’s model of health literacy [13,14] and adapted using domain descriptions outlined by Velardo [10], conceptualizing NL as a multidimensional construct encompassing functional, interactive, and critical domains. The objectives of this study were 3-fold as follows: *1*) to identify and synthesize shared NL definitions, themes, and indicators reported in the peer-reviewed literature; *2*) to construct a concept map of common NL domains and subdomains via thematic analysis; and *3*) to develop an evidence-based conceptual framework reflecting the multidimensional structure of NL among United States adults. By establishing a standardized foundation, this work seeks to advance theoretical clarity, strengthen measurement consistency, and support the development of comprehensive NL assessment tools.

## Methods

### Review protocol

#### Study design

This study integrated qualitative and quantitative components across 2 phases: *1*) framework synthesis and *2*) framework refinement. In the first phase, a scoping review and concept analysis were conducted to synthesize existing definitions, frameworks, and measures of NL among adults aged ≥18 y. Findings were organized within Nutbeam’s functional, interactive, and critical literacy domains to form an initial conceptual model. In the second phase, the preliminary framework was refined through structured expert consultation using mixed-method feedback (quantitative ratings and qualitative comments) to enhance conceptual clarity, coherence, and practical applicability. This iterative, multiphase design facilitated the mapping of existing evidence, followed by validation through expert input, improving translational relevance.

#### Framework mapping

##### Scoping review

This study followed the scoping review stages by Arksey and O’Malley [26], including considerations by Levac et al. [27], and was guided by the Joanna Briggs Institute recommendations for scoping reviews and the PRISMA extension for scoping reviews checklist. Ethics approval and registration was not required for this study [28]. The primary research questions guiding this review were as follows: *1*) what concepts, frameworks, or conceptual models exist for NL? and *2*) how do they incorporate the dimensions of functional, interactive, and critical NL?

##### Eligibility criteria

Studies were included if they focused on adults aged ≥18 y, including adults with chronic disease, and examined NL frameworks, models, programs, interventions, assessment tools, or reviews such as systematic reviews, scoping reviews, or meta-analyses. Studies were excluded if they focused on children or adolescents, addressed food literacy without distinction from NL, combined food and NL into a single construct, or measured nutrition knowledge only. The term food literacy was included as a search term to ensure that NL publications using food literacy in the title, while maintaining conceptually distinct constructs, were not overlooked. All geographic regions were included to capture the full range of existing NL frameworks, recognizing that research in this area is more advanced in some countries; this broader inclusion allowed for the identification of key themes and approaches that could inform and be adapted to a United States-focused framework. Additional exclusions included observational studies without tool or intervention development, crosscultural validation studies, protocols, editorials, books, letters, dissertations, and theses. No date restrictions were applied to ensure comprehensive capture of the evolution of NL concepts and frameworks over time, including foundational and more recent publications relevant to informing the current study.

#### Data sources and search strategy

The electronic databases of PubMed, Web of Science, CINAHL, and Google Scholar were searched for potentially eligible articles. Search terms included “nutrition literacy,” “nutritional literacy,” or “food literacy.” Only peer-reviewed journal articles published in English were considered, and no geographic restrictions were applied. No constraints were placed on the dates of publication of studies. The search for potentially eligible articles occurred up to August 2025.

### Study selection

**FIGURE 1:**
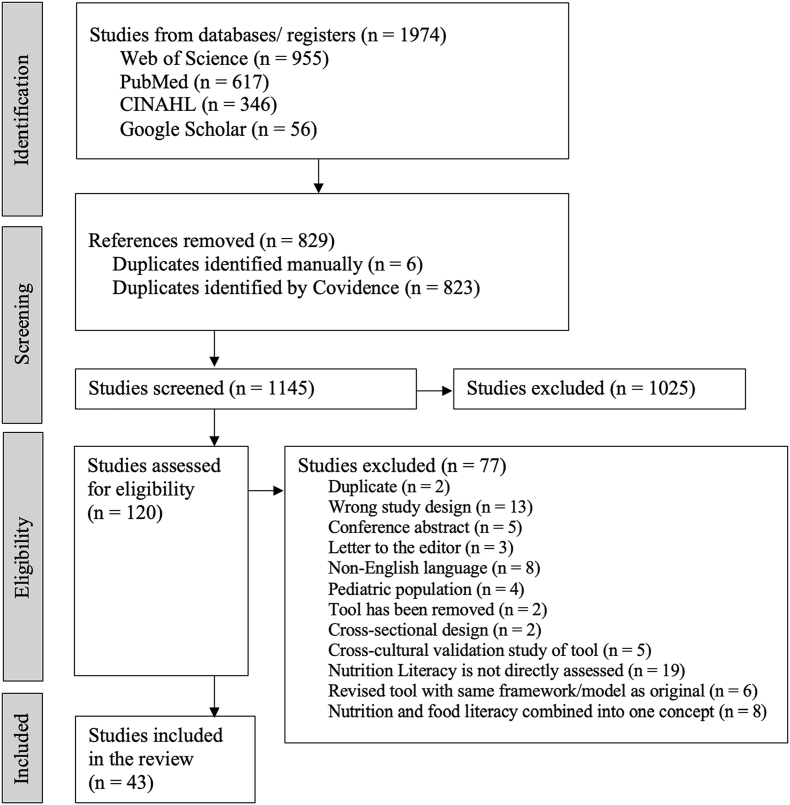
CONSORT flow diagram.

Articles were uploaded into Covidence, an online scoping review platform for screening. Reference lists of included studies and relevant reviews were also screened for additional eligible articles. Full-text reviews were conducted by the primary and secondary reviewers to identify studies that met inclusion criteria. Disagreements between primary and secondary reviewers warranted the inclusion of a third reviewer for consensus on given articles eligibility (Figure 1).

### Data extraction, synthesis and thematic analysis

A data extraction template created within Covidence was used for the collection of data focused on study characteristics (eg first author, year of publication, and geographical location), definitions of NL, use or development of conceptual frameworks or models, descriptions of programs or interventions, development and validation of assessment tools, and reported outcomes. Two reviewers independently screened all records and extracted data from the included studies. Any discrepancies were resolved through discussion until consensus was reached.

**FIGURE 2:**
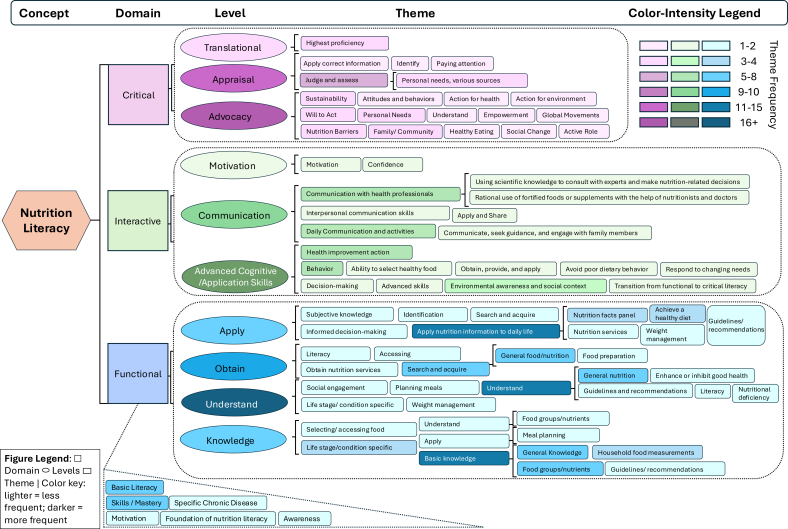
Nutrition literacy concept map.

The scoping review process concluded with collating, summarizing, and reporting the results, consistent with the framework by Arksey and O’Malley [26]. To assess NL themes and determinants in each study, a thematic analysis was conducted, emphasizing how definitions and frameworks incorporated the 3 domains of NL. Each definition was segmented into components and color-coded by 2 reviewers to identify thematic clusters. Subdomains were then identified to capture variation within each domain, and these served as indicators in the thematic analysis, contributing to a concept analysis approach. This process facilitated the development of a synthesized conceptual framework for NL by integrating overlapping components across existing definitions, models, and indicators, creating a quantitative weighted thematic analysis data set for functional, interactive, and critical NL. Supplemental Tables 1–3 were used to create a concept map of NL (Figure 2).

### Expert review: consult with stakeholders

Consistent with Arksey and O’Malley [26], a consultation exercise was incorporated to enhance validity and real-world applicability. This approach positioned expert feedback as an essential component of definition and concept development and bridging academic synthesis with applied relevance. Seven content experts were purposively recruited based on advanced training, including 4 doctoral and 3 MS, Registered Dietitians with professional experience in nutrition, public health, and/or health literacy. Experts were provided with the preliminary framework, domain definitions, and level examples and completed a structured 25-item Qualtrics survey (Supplemental Methods). The survey included background items (degree, credentials, professional role, and years of experience) followed by detailed Likert-scale and open-ended questions evaluating the clarity, coherence, and comprehensiveness of the framework. Likert-scale items (1 = strongly disagree to 5 = strongly agree) assessed *1*) overall framework organization and application potential, *2*) domain definition clarity, and *3*) alignment of level examples with definitions. Open-ended responses elicited feedback to improve conceptual precision and practical usability. Quantitative data were summarized descriptively, and qualitative responses underwent review to identify areas for refinement. All 7 experts completed the consultation survey.

## Results

### Thematic analysis and concept map

The literature search through Web of Science (955), PubMed (617), CINAHL (346), and Google Scholar (56) identified 1974 records, of which 829 duplicates were removed (Figure 1). The remaining 1145 titles and abstracts were screened by a primary and secondary reviewer to consider including in the full-text review process, resulting in 120 articles for full-text review. A total of 43 peer-reviewed journal articles were included in the scoping review.

Study, intervention, and sample characteristics of all studies were included in the review (Supplemental Methods). Of the included studies, most were conducted in China (*n* = 11, 26%) and the United States (*n* = 10, 23%), followed by Taiwan (*n* = 3, 7%). Additional studies were conducted in India, Portugal, Indonesia, and Iran (each *n* = 2, 5%), with single studies (*n* = 1, 2% each) from Austria, Belgium, Canada, Chile, Greece, Japan, Norway, and Palestine (Supplemental Tables 4–7).

Thirty-seven (86%) articles defined NL, 12 (28%) included an NL program or intervention, 18 (42%) focused on assessment tool development or validation, and 11 (26%), 9 (21%), and 3 (7%), used existing, adapted, or original frameworks/conceptual models, respectively. Framework and conceptual models were adapted from several sources including Nutbeam’s tripartite model of health literacy [13] used in 15 (35%) articles; Velardo’s domains [29] used in 2 (5%) articles; a 5-domain indicator system to assess NL for Taiwan college students [10] in 2 (5%) articles; and NL core items for Chinese lactating women [30] used in 2 (5%) articles. Several additional models were each used once, including the knowledge, attitude, and practice theory [29]; a conceptual framework of NL with multiple features [22,31]; concept of health literacy by Sørensen et al. [32]; the theory of knowledge, behavior, and belief [33]; core components of NL of the elderly [34]; food well-being [35]; and educating for sustainability framework [36]. Existing NL frameworks were all conducted in China and varied in their target populations and development methods (Table 1) [37,38]. The remaining articles discussed NL as a concept. Across the 30 studies that examined NL domains, the number of domains identified ranged from 1 to 13. Seven studies additionally described hierarchical levels within these domains. Study populations were diverse in age, gender, and education and included young adults/college students [20,21,23,[39], [40], [41]]; rural communities [42]; individuals with chronic conditions—end-stage kidney disease [9], gout [43], non-alcoholic fatty liver disease [44], breast cancer [44], diabetes [45], hypertension [46], and overweight/obesity [20,47]; women [48]; lactating women [25]; pregnant women [38,49,50,51]; mothers with young children [52]; older adults [15,22,37,53]; health care students [16,54]; those attending outpatient clinics [55]; and general adult populations [5,11,12,[56], [57], [58], [59], [60], [61], [62]].

**TABLE 1:** Overview of existing nutrition literacy frameworks across studies

Study characteristics	Author, year, country		
	Aihemaitijiang et al., 2022, China [37]	Zhang et al., 2022, China [24]	Zhou et al., 2022, China [38]
Target population	Chinese older adults	Chinese adults	Chinese pregnant women
Framework method	Framework was constructed by literature retrieval, group discussion, and expert consultation	Modified Delphi method was adopted to develop the survey framework with multiple features and indicators for assessing nutrition literacy. The research process was divided into 2 steps: *1*) review the literature to identify concepts of nutrition literacy and its components and then draft a framework with multiple features for measuring nutrition literacy and further develop the items pool; *2*) apply the Delphi method to gradually agglomerate the opinions of experts on nutrition literacy to form a consensus	Based on the Health Literacy of Chinese Citizens 66 Items—Basic Knowledge and Skills
Domains	Knowledge and understanding	Cognition	Basic knowledge and ideas
	Healthy lifestyle and dietary behavior	Skill	Lifestyle and dietary behaviors
			Basic skills
Domain formulation	Based on Dietary Guidelines for Chinese Residents (2016), Dietary Guidelines for the Elderly (2016), Auxiliary Reference Food Atlas of Retrospective Dietary Survey, and the National Application for 2015 China Aging and Health Assessment	Literature review	Two rounds of Delphi expert consultation and group discussion were conducted to determine the final NL core items (including domains) for Chinese pregnant women, in addition to initial framework development
	Skill	—	—
Levels	—	Functional	Basic nutrition concept
		Interactive	Food and nutrition knowledge
		Critical	Nutrition and disease knowledge
			Lifestyles
			Dietary behaviors
			Preparation for breastfeeding
			Gestational weight management
			Gestational disease management
			Acquisition
			Understanding and application of nutrition information
			Judgment of nutrition information
			Nutrition decision making
Other framework components	—	6 dimensions were included in the framework: knowledge, understanding, obtaining, applying, interactive skills, and critical skills	—
Purpose	To develop a Nutrition Literacy Questionnaire for the Chinese Elderly	To develop a conceptual framework with multiple features and further establish a nutrition literacy measurement scale for Chinese adults by using the Delphi method.	To develop the nutrition literacy assessment tool for Chinese pregnant women (NLAI-P), with the expectation of providing a unified tool for assessing and monitoring the status of Nutrition literacy of pregnant women in China

Definitions of NL domains were not consistent or were lacking across countries (Table 2). China most frequently addressed all 3 domains, followed by the United States. Other countries contributed far fewer publications. A substantial subset of articles did not explicitly define functional, interactive, or critical NL domains.

**TABLE 2:** Distribution of functional, interactive, and critical nutrition literacy domains across countries

Country of origin	Nutrition literacy domains, *n* (%)		
	Functional literacy	Interactive literacy	Critical literacy
Austria	1 (2)	—	—
Belgium	1 (2)	1 (2)	1 (2)
Chile	1 (2)	1 (2)	1 (2)
China	7 (16)	7 (16)	6 (14)
Indonesia	1 (2)	—	1 (2)
Italy	1 (2)	1 (2)	1 (2)
Norway	1 (2)	1 (2)	1 (2)
Palestine	1 (2)	1 (2)	1 (2)
Portugal	—	1 (2)	1 (2)
Switzerland	1 (2)	1 (2)	1 (2)
United States	4 (9)	3 (7)	3 (7)
Articles not defining NL domains	24 (44)	25 (58)	17 (40)

This table lists the number of studies that addressed respective domains at least once in the study.

A total of 105 NL themes were identified and quantified by frequency mentioned in the literature across the included full-text articles and are presented in Supplementary Table 2. Guided by Nutbeam’s model of health literacy and results from the thematic analysis, these themes were consolidated into an NL concept map (Figure 2), including 3 primary domains: functional, interactive, and critical literacy. Nine corresponding levels were created, including knowledge, understand, obtain, apply, advanced application, communication, motivation, appraisal, advocacy, and translational literacy.

### Functional NL definition and levels

**FIGURE 3:**
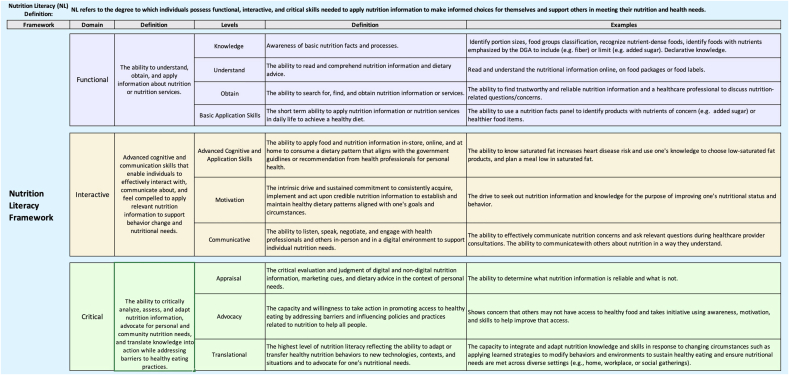
Nutrition literacy framework for adults.

Figure 3 includes functional literacy domain and level definitions with subsequent level examples. Functional literacy and subsequent levels of knowledge, understand, obtain, and apply (basic applications skills) are described or defined throughout the literature, and descriptions and definitions were modified and implemented in accordance with the concept map and Velardo’s viewpoint [10]. Functional literacy is defined as the ability to understand, obtain, and apply information about nutrition [12,15,24,41,43,49] or nutrition services and is referred to as the foundation of NL [15,45]. Knowledge is defined as awareness of facts and processes or as basic nutrition knowledge [9,[23], [24], [25],^38^,^63^]. Understand is defined as the ability to read and comprehend nutrition information [12,21,39,58,63] and dietary advice [23]. Obtain is defined as the ability to search for, find, and obtain nutrition information or services [9,12,21,23,24,39,58]. Apply, modified to basic application skills in accordance with expert feedback, is defined as the short-term ability to apply nutrition information or nutrition services in daily life [18,21,25,31,40,55,56,58] to achieve a healthy diet [21,23,24,39]. New levels were identified within interactive (advanced application, motivation, and communication) and critical (appraisal, advocacy, and translational) literacy domains, reflecting a more nuanced progression of skills and capacities across the continuum of NL.

### Development of interactive and critical NL level definitions

The following interactive and critical NL domain level definitions were formulated through synthesis of the scoping review findings, thematic analysis, and concept mapping (Figure 2) and established theoretical perspectives, including Velardo’s viewpoint [10] and Nutbeam’s health literacy model [12]. In brief, interactive NL is the transition from functional to critical literacy [15] defined as advanced cognitive and communication skills that enable individuals to effectively interact with, communicate about [16,20,41,49], and feel compelled to apply relevant nutrition information to support behavior change [45] and nutritional needs [5,11,12,15,23,24,40,43,63]. Critical NL, the highest level of proficiency [5,15,16,20], is defined as the ability to critically analyze [42], assess, and adapt nutrition information [23]; advocate for personal and community nutrition needs; and translate knowledge into action [5,11,12] while addressing barriers to healthy eating practice [15,16,20,38,40,41,43,49,60,64,65]. While all domains and levels were informed by the full body of evidence, certain definitions emphasize specific studies where conceptual support was strongest. References are provided to reflect primary sources informing each level.

#### NL advanced cognitive and application skills

At a basic level, advanced cognitive and application skills are interactive NL application skills that reflect an ability to translate declarative knowledge into positive dietary choices [12] in various scenarios [63] including when at a store, online, or at home [25,51]. Examples of lifestyle and dietary behavior reflective of advanced cognitive and applications include meal-timing, chrononutrition [37], interpretation of nutrition labels, weight management, and nutrition information decision making according to one’s specific life stage or disease state [25]. Therefore, based on the existing literature, we propose the level of NL advanced cognitive and application skills as the ability to apply food and nutrition information in-store, online, and at home to consume a dietary pattern that aligns with the government guidelines or recommendations from health professionals for personal health or health of others.

#### Motivational NL

Motivation, in relation to NL, is described as skills and confidence to navigate the food system and the capacity to apply nutrition information to improve personal health skills and status [12]. Velardo’s viewpoint [10] emphasizes its role in sustaining engagement with health information and supporting ongoing behavior change [42]. Furthermore, Sørensen et al. [32] present an integrative model of health literacy that explicitly names motivation as an important component to knowledge and competencies. Therefore, based on existing literature on NL motivation, we propose the level of motivational NL as the intrinsic drive and sustained commitment to consistently acquire, implement, and act upon credible nutrition information to establish and maintain healthy dietary patterns aligned with one’s goals and circumstances.

#### Communicative NL

Communicative NL [15,20,21,37,43,49] is commonly adapted from Nutbeam’s definition of communicative literacy [13], advanced cognitive and literacy skills, which, together with social skills, can be used to participate in everyday activities to extract information and derive meaning from different forms of communication and to apply new information to changing circumstances. The communicative level is discussed as the ability to communicate, seek guidance, and engage with health professionals [16]/family members [49] and share information [15] to meet personal daily needs [21,43]. Therefore, we propose the level of communication NL as the ability to listen, speak, negotiate, and engage with health professionals and others in-person and in a digital environment to support individual nutrition needs.

#### Appraisal NL

Appraisal related to NL is commonly addressed in the literature [11,16,21,40,43,63] as the capacity to judge and assess nutrition information in terms of personal needs. This includes assessing the credibility of sources (eg distinguishing credible from noncredible information) [40]; identifying nutrition myths or misleading claims (e.g. carbohydrates cause weight gain or detox diets remove toxins) [21]; and evaluating the quality of evidence (eg accuracy, completeness, and level of scientific support) [11,43]. Authors also describe appraisal as determining the validity and applicability of nutrition information and advice from various sources for personal use [16,63]. Therefore, we propose the level of appraisal NL as the critical evaluation and judgment of digital and nondigital nutrition information, marketing cues, and dietary advice in the context of personal needs.

#### Advocacy NL

Advocacy related to NL emphasized the significance of the capacity to act on and influence the underlying social determinants of health, further suggesting advocacy as one of the most advanced levels of nutrition or health literacy [12,16,51,63]. Critical NL should consequently encompass and increased awareness and critical/emancipatory action to address barriers to good nutrition [16] challenging deeply engrained sociocultural norms regarding nutrition and health [10]. Therefore, we define advocacy NL as the capacity and willingness to take action to promote equitable access to healthy eating by analyzing and addressing systemic inequities in food access, including disparities in availability and affordability; engaging in policy processes (eg food distribution and subsidy structures); and influencing practices and environments through community and civic action.

#### Translational NL

Translational literacy was based off of NL literature [21] and derived from the transactional model of communication, in which translational eHealth literacy is described as the highest cognitive level of eHealth literacy and the ability to apply health knowledge gained from the internet across diverse ecological contexts [66]. Therefore, developing a concept that acknowledges the dichotomy between knowledge and action represents a gap in the NL literature. In public health research, knowledge translation is a systems-level approach to transforming knowledge acquired to ameliorated health outcomes [67]. Characteristics of NL reflecting the translational level were described as a level translating to the ability to use their nutrition knowledge and skills to apply nutritional information to one's specific context and needs [49] to acquire control over their life events [5]. Therefore, we propose the level of translational NL be defined as the highest level of NL reflecting the ability to adapt or transfer healthy nutrition behaviors to new technologies, contexts, and situations and to advocate for one’s nutrition and dietary needs.

**FIGURE 4:**
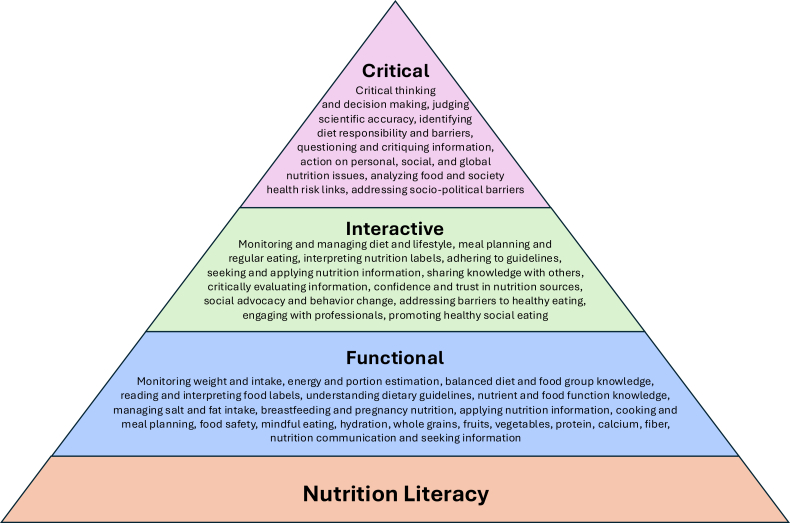
Conceptual pyramid of nutrition literacy domains and determinants.

To define the determinants of each domain and relationships among domains and their defining competencies, a conceptual pyramid (Figure 4) was developed to represent the NL framework determinants. The pyramid depicts functional, interactive, and critical literacy as interconnected and progressive domains, with corresponding indicators reflecting an individual’s competence within each domain.

### Framework adaptation phase

Agreement ratings across the 51 expert consultation items demonstrated strong overall consensus, with means ranging from 4.6 to 4.8 and SDs ranging from 0 to 1.3, with experts commenting that the NL framework was conceptually coherent, logically structured, and applicable to both research and practice. Item-level means, SDs, and comments are reported in Supplemental Materials. A small number of items received lower ratings from ≥1 expert, including 4 items that scored as low as 3 related to the interactive and critical literacy domains and motivation definition clarity and 1 item that scored as low as 2, assessing alignment between the “understand”-level examples and its definition.

Qualitative feedback converged on 3 major themes: *1*) clarification of domain and level distinctions, *2*) refinement of terminology and examples, and *3*) guidance for applied use. Experts emphasized the need to better delineate overlapping levels within and across domains. One reviewer noted, “It can be challenging to distinguish between advanced cognitive and application skills and the apply level—more examples could help clarify the differences.” Another highlighted the similarity between advocacy and translational literacy, suggesting “translation might be more about applying nutritional understanding in different contexts, whereas advocacy focuses on encouraging change among friends, family, or the broader food environment.”

Terminology refinement was also a recurrent theme. Reviewers recommended adjusting phrasing to improve clarity and accessibility—for instance, shortening definitions for readability. As 1 expert wrote, “The ability to understand, obtain, and apply … nutrition services doesn’t fully make sense—maybe say ‘apply information about nutrition or nutrition services.’” Another observed that critical literacy might be interpreted differently by readers from educational disciplines, recommending a potential name revision to avoid confusion with “critical pedagogy.”

Reviewers encouraged stronger integration of motivation and communication within the interactive domain, describing these as “dynamic processes that connect understanding to behavior change.” Several experts also recommended providing dual examples, one for individual assessment and another for intervention planning to demonstrate versatility. One summarized, “This framework could be used to create interventions ensuring that multiple domains are addressed—like including Registered Dietitian Nutritionists skilled in motivational interviewing to address the motivation level.”

Following incorporation of these revisions, the principle features of the NL framework (Figure 3) were 3 interrelated domains (functional, interactive, and critical literacy), progressing across 9 levels (knowledge, understand, obtain, apply, advanced cognitive application, communication, motivation, appraisal, advocacy, and translational) of skill and cognitive development. Domain and level definitions and examples can be found in Figure 3. These levels expressed the basic meaning of NL. NL definitions grouped by country of origin are found in Supplementary Tables 4–7. The final unified NL sramework establishes a comprehensive definition derived from thematic analysis and expert feedback.

### Definition of NL

NL refers to the degree to which individuals possess functional, interactive, and critical skills needed to apply nutrition information to make informed choices for themselves and support others in meeting their nutrition and health needs.

## Discussion

This scoping review synthesizes how NL has been defined, conceptualized, and operationalized across adult populations in the United States and internationally. Across the 43 included studies, we identified substantial variability in terminology, domain structure, and assessment approaches, with many articles addressing only isolated components of functional, interactive, or critical literacy and a considerable proportion lacking explicit domain definitions. Through thematic analysis, 105 unique themes were identified and consolidated into a conceptual map that organized NL into 3 domains—functional, interactive, and critical literacy—and 9 corresponding levels. These levels clarified competencies that were well established in the literature (eg knowledge, understand, obtain, and apply), as well as those inconsistently defined or newly emerging (eg advanced cognitive and application skills, communication, motivation, appraisal, advocacy, and translational literacy). Guided by these findings and expert feedback, we developed a multidomain, multilevel NL framework that integrates Nutbeam’s model with existing NL conceptualizations and emergent skills identified in the review. This framework offers a unified structure for improving conceptual alignment and supporting future measurement, research, and intervention design in the United States.

International efforts to conceptualize NL have been limited, with only 3 published frameworks to date, each developed in China and designed for specific populations: older adults [37], general adults [24], or pregnant women [38]. As summarized in Table 1, these models relied on literature review, expert consultation, or Delphi processes and were largely constructed to develop population-targeted assessment tools rather than establish broad conceptual frameworks. Their domain structures frequently relied on national dietary guidelines, with competencies centered on foundational knowledge, cognition, and basic dietary behaviors. While these frameworks are valuable within their intended populations, their scope is narrow, population specific, and not fully aligned with broader conceptualizations of NL as a form of health literacy.

Across these frameworks, levels of skill development were not consistently defined, and interactive and critical capacities, such as communication, appraisal, or decision making, were variably represented or embedded implicitly within broader categories. As shown in Table 2, articles from the United States focused only on functional literacy. Additionally, 44% of studies did not define NL domains at all, further highlighting conceptual fragmentation. There have been calls to move beyond assessment of functional NL to define and capture sociocultural domains that influence and formulate NL [10]. Following this publication, antecedents of NL have been identified as action verbs and concepts such as to grasp the essence, to follow, to problem solve, to make right food choices, how 1 interacts with nutrition information guidelines and advice, and the concept of healthful diets [65]. These emergent themes can inform the development of distinct levels within the interactive and critical domains, allowing for greater conceptual precision and alignment with real-world nutrition practices.

### NL advanced cognitive and application skills

Across the literature, advanced cognitive skills and application emerged as underdeveloped components of NL despite their central relevance to real-world dietary decision making. Velardo [10] describes interactive NL is a person’s ability to use nutrition knowledge in a meaningful way and within the social, cultural, and environmental contexts of their life, which largely describes the advanced application level of our thematic analysis. Other studies fail to define the advanced cognitive and application domain but acknowledge the importance of perceived ability to make healthful eating decisions in differing scenarios, such as budgeting or planning meals [63]. Many studies focused narrowly on knowledge acquisition or basic understanding, with few addressing higher-order abilities such as interpreting complex nutrition information, integrating competing messages, or adapting recommendations to personal contexts. Indicators of advanced application such as modifying dietary patterns based on health needs, evaluating the appropriateness of food choices, or translating recommendations into daily practice were inconsistently represented and often embedded within broader functional constructs. These gaps highlight the need for clearer delineation of advanced cognitive and application skills, separate from critical literacy, within NL frameworks, as these competencies underpin the skills to act on information in meaningful and sustained ways.

### Communicative NL

Although communication is a cornerstone of Nutbeam’s health literacy model [14], few NL studies explicitly assessed the ability to engage in dialog about nutrition information, seek clarification, or exchange information with health professionals [20,37], peers, or family members [12]. Strengthening communication as a clearly defined level within the interactive domain is essential, as individuals must be able to articulate concerns, negotiate dietary decisions, and participate in interpersonal exchanges that influence food choices [10].

### Motivational NL

Motivation has long been recognized as a component of health literacy. Nutbeam and Kickbusch [68] originally described health literacy as the cognitive and social skills, which determine the motivation and ability of individuals to gain access to, understand and use information in ways that promote and maintain good health. Similarly, Sørensen et al. [32] linked health literacy to people’s knowledge, motivation, and competencies to access, understand, appraise, and apply health information in daily health decision making [32]. Despite this emphasis in health literacy theory, motivation is rarely included when NL is conceptualized or measured. In 2015, Velardo [10] stated complex skills, motivation, and confidence needed to navigate the food system should be prioritized. A 2018 systematic review of nutrition and food literacy measurement tools [12] identified only limited attention to motivational processes, noting that interactive literacy rarely included skills, motivation, and confidence to navigate the food system. One NL tool, the critical NL instrument, addressed this criterion, which is a 19-item instrument measuring the following 2 domains: engagement in dietary habits and taking a critical stance toward nutrition claims and their sources [16]. However, the construct captured reflects confidence rather than motivation. Motivation remains largely absent from NL models and assessment tools. Given its role in broader health literacy frameworks, motivation should be explicitly incorporated into NL to more accurately capture potential behavioral drivers that influence dietary decision making.

### Appraisal NL

Appraisal, a key component of critical literacy, was consistently represented in the literature that addressed the domain of critical literacy [40,58,63,64,69]. Some studies assessed whether individuals could evaluate the quality of nutrition information, identify misleading claims, or compare sources, but these skills were often embedded within broader critical NL domain rather than conceptualized as a distinct level. As misinformation proliferates in digital and commercial food environments, the ability to critically evaluate nutrition information is increasingly essential. Strengthening appraisal as an independent level will support better alignment with health literacy models and improve the field’s capacity to study how individuals navigate complex and sometimes contradictory nutrition messages.

### Advocacy NL

Advocacy emerged as one of the least developed components of NL in the existing literature, despite its central role in critical NL presented by Velardo [10]. Across studies, few explicitly examined individuals’ capacity to articulate their dietary needs, request accommodations, or influence household, community, or institutional food environments. When advocacy was mentioned, it was typically embedded within broader critical literacy constructs rather than treated as a distinct competency. However, with the development of an NL tool for young adults, a theme of advocacy for a healthful food environment was identified [63]. Young adults can be effective catalysts of change, but college students report barriers with accessing resources, organizing efforts, and getting people to listen [64]. Therefore, given its theoretical grounding and practical relevance, advocacy warrants explicit inclusion within NL frameworks to capture higher-order capacities that extend beyond individual knowledge and behavior to the broader social and environmental contexts that influence personal dietary choices and the willingness to take action to help others.

### Translational NL

Translational literacy, the most advanced level in our framework, remains underdeveloped within existing NL research. Although the term is rarely used in the nutrition literature, Liao and Lai [21] described apply as the capacity to use nutrition information in daily life to promote a healthy diet, a concept closely aligned with translational literacy. Indicators of this level include modifying dietary patterns based on personal health needs, limiting highly processed foods, and selecting plant-based options. A systematic review of 40 literacy models similarly defined translational literacy as the ability to adapt or transfer health-related behaviors to new contexts, technologies, and situations and to advocate for one’s nutrition and dietary needs [70]. Together, these characteristics reflect an advanced capacity to integrate nutrition information into sustained action. However, despite its relevance for real-world dietary decision making, translational literacy remains largely unexamined and requires further conceptual development in future research.

### Strengths and limitations

Our study was strengthened by its systematic approach to the literature search, thematic analysis, and expert feedback. The framework is additionally strengthened by its foundation in Nutbeam’s widely accepted model of functional, interactive, and critical health literacy. Several authors have referenced Nutbeam’s model, which has been used as an analytical grid in multiple studies and recommended as a tool to map different skills and abilities [10]. Although this scoping review and thematic analysis examined the concept of NL and proposed a conceptual framework through thematic analysis, concept mapping, and expert review, it is not without limitations.

First, NL and food literacy have long coexisted in the literature, yet the boundaries between them remain unclear. This overlap has made it difficult to measure effects or compare the efficacy of interventions focused on NL. For this reason, we intentionally focused on literature that clearly differentiated NL from food literacy to clarify current uncertainties surrounding the concept and components of NL. However, because some literature conceptualizes NL as a subset of food literacy, information relevant to NL found within food literacy publications may have been excluded, although such material could meaningfully contribute to framing NL as a form of health literacy [11]. Additionally, included United States studies represented heterogeneous populations, and NL competencies may vary across cultural, socioeconomic, or linguistic groups not fully captured in the available literature. Finally, our search was limited to peer-reviewed literature indexed in the selected databases, which may have excluded relevant gray literature or emerging models not yet published.

### Future research

Given the central role of nutrition in daily life and its importance in the prevention and management of chronic disease, NL, as a specific form of health literacy, has the potential to meaningfully inform future health promotion efforts focused on dietary behavior. Findings from this review indicate that substantially more research is needed on interactive and critical literacy skills, which remain underdeveloped compared with functional literacy skills. Future work should also examine the roles of attitudes, motivation, and behavior, which emerged prominently in our analysis but have not been fully integrated into existing NL models. These components remain points of debate in the broader health literacy field and require further conceptual clarification and empirical testing within nutrition contexts. Additionally, quantitative data on NL are limited, underscoring the need for studies that provide stronger empirical support for its role as a determinant of health and well-being.

Although our NL framework was developed to organize and assess domains and levels across the literature, it also provides practical direction for program, intervention, and assessment tool development. Importantly, professionals in dietetic and nutritional fields have a key role in positively influencing NL, because they disseminate evidence-based information to patients during doctor appointments. For example, incorporating registered dietitian nutritionists trained in motivational interviewing to support the motivation level of the interactive domain. Such applications may help practitioners move beyond functional knowledge to strengthen interactive and critical competencies. Future research should test how this framework can be applied in both individual assessment and intervention design to improve health behavior outcomes. There is a clear need for assessment tools that capture all domains and levels of the framework, assess test–retest reliability, provide direction for scoring, and include both subjective and objective measures [12]. Furthermore, many United States instruments, specifically the NLit, with the strongest psychometric properties, remain focused on functional literacy [12].

This review also highlights broader research considerations. United States efforts have typically emphasized functional skills, with limited attention to the interactive competencies or critical capacities required to navigate complex food environments. A multidimensional framework can help clarify which components of NL are being targeted in research and practice. When comprehensive approaches are not feasible, practitioners may specify the particular domains or levels they intend to address to enhance alignment between program goals and literacy outcomes. Future studies should also investigate structural, social, and cultural influences on NL and how these contextual factors shape the development and use of literacy skills. Mixed-methods and qualitative approaches may be particularly valuable for examining how individuals interpret and apply nutrition information in real-world settings in the United States.

## Conclusions

In summary, we developed a multidomain, multilevel conceptual NL framework for adults living in the United States. Our scoping review and thematic analysis revealed areas of concentrated attention in the literature, particularly functional literacy and knowledge-based competencies, as well as notable gaps across interactive and critical domains and their corresponding levels. These findings highlight key opportunities for advancing conceptual clarity, strengthening measurement, and enhancing NL research and intervention design.

## Author contributions

The authors’ responsibilities were as follows—ALH, EKH, XL, JIB: designed research; ALH, SB, JIB, SS, KSS: conducted research; ALH, SB: analyzed data; ALH, SB: wrote the paper; ALH: had primary responsibility for final content; and all authors: have read and approved the final manuscript.

## Data availability

Data described in the manuscript, code book, and analytic code will be made available upon request pending application.

## Declaration of Generative AI and AI-assisted technologies in the writing process

The authors declare that no generative AI or AI-assisted technologies were used in the writing of this manuscript.

## Funding

This study was funded by the USDA National Institute of Food and Agriculture, Agriculture and Food Research Initiative (AFRI; grant 2023-68015-39408).

## Conflict of interest

The authors report no conflicts of interest.
