# Supplementary material for: A Conceptual Nutrition Literacy Framework for Adults in the United States: A Scoping Review and Thematic Analysis
Source: Adv Nutr. 2026 Jun 18;17(8):100686. doi: 10.1016/j.advnut.2026.100686 (PMC13382296; doi:10.1016/j.advnut.2026.100686)
Supplement: Multimedia component 1 [file mmc1.docx]

**Supplementary Table 1.** Function Nutrition Literacy Weighted Themes Across 43 studies including Knowledge, Understand, Obtain, and Apply Levels.

| **Theme** | **Count** | **Determinants** | **Count** |
| --- | --- | --- | --- |
| Functional Domain |  |  |  |
| Understand | 5 |  |  |
| Obtain | 10 | Utilize nutrition services |  |
| Knowledge | 5 |  |  |
| Apply | 7 |  |  |
| Awareness | 1 |  |  |
| Foundation of NL | 2 |  |  |
| Specific Chronic Disease | 2 |  |  |
| Skills/Mastery | 5 |  |  |
| Motivation | 1 |  |  |
| Basic Literacy | 5 |  |  |
| Level: Knowledge |  |  |  |
| Understand | 1 | Food Groups/Nutrients | 1 |
| Life stage/ condition specific | 3 |  |  |
| Basic Knowledge | 12 | General | 7 |
|  |  | Macronutrients | 2 |
|  |  | Household food measurements | 3 |
|  |  | Guidelines /Recommendations | 2 |
|  |  | Food Groups/Nutrients | 5 |
|  |  |  |  |
| Selecting/Accessing food | 1 |  |  |
| Apply | 1 | Meal planning | 1 |
| Level: Understand |  |  |  |
| Understand | 13 | Nutritional Deficiency | 1 |
|  |  | Literacy | 1 |
|  |  | General Nutrition | 8 |
|  |  | Guidelines and Recommendations | 2 |
|  |  | Enhance or Inhibit Good Health | 1 |
| Life stage/ condition specific | 1 |  |  |
| Weight Management | 1 |  |  |
| Planning Meals | 1 |  |  |
| Social Engagement | 1 |  |  |
| Level: Obtain |  |  |  |
| Search and Acquire | 7 | General food/nutrition | 6 |
|  |  | Food preparation | 1 |
| Obtain Nutrition Services | 2 |  |  |
| Accessing | 1 |  |  |
| Literacy | 1 |  |  |
| Level: Apply |  |  |  |
| Apply nutrition information to daily life | **11** | Weight Management | 1 |
|  |  | Nutrition Services | 2 |
|  |  | Achieve a healthy diet | 3 |
|  |  | Guidelines/Recommendations | 1 |
|  |  | Nutrition Facts Panel | 4 |
| Identification | 1 |  |  |
| Informed decision-making | 2 |  |  |
| Search and acquire | 1 |  |  |
| Subjective Knowledge | 1 |  |  |

*Specific level themes are designated if manuscript specifically described this level as its own component of nutrition literacy.

**Supplementary Table 2.** Interactive Nutrition Literacy Weighted Themes Across 43 studies including Advanced Cognitive and Application, Motivation, and Communication levels.

| **Theme** | **Count** | **Specific Topics** | **Count** |
| --- | --- | --- | --- |
| **Interactive Domain** |  |  |  |
| Domain Transition | 1 |  |  |
| Advanced Cognitive and Application Skills | 11 | Health improvement action | 5 |
|  |  | Decision-making | 2 |
|  |  | Behavior | 3 |
|  |  | Obtain, provide, and apply | 1 |
|  |  | Advanced skills | 2 |
|  |  | Respond to changing needs | 1 |
|  |  | Avoid poor dietary behavior | 1 |
|  |  | Environmental awareness and social context | 3 |
| Communication | 10 | Apply and share | 1 |
|  |  | Communicate, seek guidance and engage with family | 1 |
|  |  | Daily communication and activities | 4 |
|  |  | Communication with health professionals | 3 |
|  |  |  |  |
| Level: Advanced Cognitive and Application Skills |  |  |  |
| Select healthy food | 1 |  |  |
| Behavior | 2 |  |  |
|  |  |  |  |
| Level: Motivation | 1 |  |  |
| Motivation | 1 |  |  |
| Confidence | 1 |  |  |
| Level: Communication |  |  |  |
| Communication with health professionals | 2 | Using scientific knowledge to consult with experts and make nutrition-related decisions | 1 |
|  |  | Rational use of fortified foods or supplements with the help of nutritionists and doctors | 1 |
| Interpersonal communication skills | 1 |  |  |

*Specific level themes are designated if manuscript specifically described this level as its own component of nutrition literacy.

**Supplementary Table 3.** Critical Nutrition Literacy Weighted Themes Across 43 Studies including Appraisal, Advocacy, and Translational Levels.

| **Theme** | **Count** | **Specific Topics** | **Count** |
| --- | --- | --- | --- |
| **Critical Domain** |  |  |  |
| Appraisal | 15 |  |  |
| Translational | 3 |  |  |
| Highest proficiency | 3 |  |  |
| Advocacy | 23 | Nutrition Barriers | 4 |
|  |  | Will to Act | 4 |
|  |  | Family/Community | 3 |
|  |  | Healthy eating | 1 |
|  |  | Sustainability | 4 |
|  |  | Attitudes and behaviors | 1 |
|  |  | Social Change | 1 |
|  |  | Empowerment | 1 |
|  |  | Understand | 2 |
|  |  | Global Movements | 1 |
|  |  | Active Role | 1 |
| **Level: Appraisal** |  |  |  |
| Judge and assess | 8 | Personal Needs | 3 |
|  |  | Various sources | 1 |
| Paying attention | 1 |  |  |
| Identify | 2 |  |  |
| Apply correct information | 1 |  |  |
| **Level: Advocacy** |  |  |  |
| Action for environment | 1 |  |  |
| Action for health | 1 |  |  |
| **Level: Translational** |  |  |  |
| None |  |  |  |

*Specific level themes are designated if manuscript specifically described this level as its own component of nutrition literacy.

**Supplementary Table 4.** Characteristics of Nutrition Literacy Studies Conducted in China, Including Study Design, Target Population, Definitions, and Sample Demographics

| **Author, Year Paper Published** | **Country** | **Study Design** | **Target**  **Population** | **Nutrition Literacy Definition** | **Age**  **(mean ± SD)** | **Gender (n)** | **Education Level (n)** |
| --- | --- | --- | --- | --- | --- | --- | --- |
| China | | | | | | | |
| Aihemaitijiang, 2022 ^1^ | China | Validation paper | Adults > 60 years (n= 1490) | An individual’s ability to obtain, process and understand basic nutrition information and use these abilities to make appropriate nutrition decisions and obtain nutrition services^2,3^. | 68.60 ± 5.543 | Male: 314, female : 1176 | Illiterate: 43, Did not finish primary school: 160, graduated from primary: 216, lower middle school degree: 571, upper middle school degree: 393 , technical or vocational degree: 83, bachelor’s degree or above: 24 |
| Li, 2023 ^4^ | China | Validation paper | Chinese lactating women (n=622) | The ability of individuals to obtain, understand and process nutritional information or services and to use information and services to make correct decisions so as to maintain and promote their own health^2^. | ≥ 18 years old | All female | High school or lower: 51, college: 116, university: 286, postgraduate and above: 169 |
| Li, 2023 ^5^ | China | Validation study | Patients with end-stage kidney disease (ESKD) receiving dialysis (n=208) | Capability of individuals to obtain and understand nutrition facts panel information, make accurate decisions, and use nutrition facts panel information to maintain and promote the nutritional status of themselves and others^6^. | n (%); <50: 93 (44.7) 51-60: 67 (32.2) >61: 48 (23.1) | Male: 124, female: 84 | - |
| Li, 2024 ^7^ | China | Randomized controlled trial | Pregnant women (n=88) | - | 26.89 ± 3.47;  26.14 ± 2.33 | All female | Below associate's degree: 24, associate degree and bachelor's degree: 56, bachelor's degree and Above: 8 |
| Li, 2025 ^8^ | China | Randomized controlled trial | Women who were 12 weeks pregnant (n=88) | Nutrition literacy is not merely knowledge of dietary guidelines; it encompasses the ability to access, understand, evaluate, and apply nutrition information in everyday contexts. | 26.14 ± 2.33; 26.89 ± 3.47 | - | Below associate degree: 24, associate/bachelor’s degree: 56, above bachelor’s degree: 8 |
| Liu, 2023 ^9^ | China | Cross-sectional study | Permanent residents of Hangzhou City | The ability of individuals to acquire, understand, evaluate and apply nutritional information to make appropriate nutritional decisions that promote health^2^. | ≥ 60 years | Male: 339, female: (631) | Primary school and below: 506, junior high school: 245, high school/ vocational school: 135, college and above: 84 |
| Mo, 2022 ^10^ | China | Validation paper | Young adults ≥ 18 years old (n=1,359) | The degree to which an individual has the capacity to obtain, process, and understand nutrition information and services required for making appropriate nutrition decisions^2,11,12^. | 22.22 ± 2.94 | Male: 553, female: 806 | Freshmen: 356, sophomores: 504, juniors: 230, seniors: 269 |
| Zhang, 2022 ^13^ | China | Validation paper | The panel of experts from China (n=15 experts) | The degree to which individuals can obtain, process, and understand nutrition information and services needed for making appropriate nutrition decisions^12,14,15^. | 45.5 | - | Bachelor’s degree: 3, master’s degree: 5, PhD: 7 |
| Zhang, 2025 ^16^ | China | Validation Study | Patients with type 2 diabetes mellitus (n= 576) | Ability of an individual to acquire, process, understand and apply nutrition information and skills, and to make appropriate nutrition decision^17^. | 37.79 ± 13.65 | Male: 357, female:  219 | Primary school and below: 32, junior high school: 109, jigh school or junior college: 168, colleges and above: 267 |
| Zhang, 2025 ^18^ | China | Validation Study | Gout patients in China (n= 526) | Ability of individuals to access, process and understand basic nutrition information and services, and to make informed nutrition decisions^11,19^. | 41.15 ± 10.19 | Male: 226, female: 300 | Primary school and below: 185, junior high school: 100, high school or junior college: 118, colleges and above: 123 |
| Zhou, 2022 ^20^ | China | Validation paper | Pregnant women (n=699) | The capacity to access, process, and understand the faculty nutrition information and the skills to use the information for appropriate decision making to maintain and promote health^12^. | 31.2 ± 4.1 | All female | Junior high school: 21, senior high school: 74, university: 263, MS or above- 341 |

**Supplementary Table 5.** Characteristics of Nutrition Literacy Studies Conducted in the United States, Including Study Design, Target Population, Definitions, and Sample Demographics

| **Author, Year Paper Published** | **Country** | **Study Design** | **Target**  **Population** | **Nutrition Literacy Definition** | **Age**  **(mean ± SD)** | **Gender (n)** | **Education Level (n)** |
| --- | --- | --- | --- | --- | --- | --- | --- |
| United States | | | | | | | |
| Bedoyan, 2021^21^ | United States | Validation study | U.S. college students, 18-24 years old (n=1870) | The ability to understand basic nutrition information and services needed to make appropriate nutrition decisions ^11,22,23^ . | 18.4 ± 1.0,  20.4 ± 1.7 | Male:  390, female: 1048 | - |
| Carroll, 2023 ^24^ | United States | Cross sectional study | Patients with and without Non-Alcoholic Fatty Liver Disease (n=2,938) | The ability to understand nutrition information and implement that knowledge to reach dietary goals^25^. | 51.6, 44.8 | Female (%): 48.2  female (%): 56.3 | Less than 9th grade: 3.7%, 2.4%; 9-11th grade: 7.2%, 5.5%; High school graduate/GED: 27.6%, 24.6%; Some college or AA degree: 32.4%, 29.2%; College graduate or above: 29.1%, 38.3 % |
| Gibbs, 2013  ^26^ | United States | Validation paper | Adult clients (n=134) | Nutrition literacy requires knowledge of nutrition principles and skill in food-related tasks^27^. | aged ≥18 years | - | - |
| Gibbs, 2016 ^28^ | United States | Non-randomized experimental study | Breast cancer survivors and women at high risk for breast cancer (n=18). | The degree to which individuals have the capacity to obtain, process, and understand nutrition information and skills needed in order to make appropriate nutrition decisions^12^. | 56.3 ± 8.1 | All female | ≤High school graduate: 6%, some college: 29%,  ≥bachelor's degree: 65% |
| Gibbs, 2018 ^29^ | United States | Validation paper | Adults with diabetes, hyperlipidemia, hypertension, or overweight/obesit (n=445) | The degree to which individuals have the capacity to obtain, process, and understand nutrition information and skills needed to make appropriate nutrition decisions^12^. | 54.0 ± 14.54 | Male: 119, female: 309 | High school/GED or less: 62, some college/associates degree: 163, bachelor’s degree or higher: 196 |
| Marchello, 2021^30^ | United States | Randomized controlled trial | Patients with the ability to read English. | The degree to which individuals can obtain, process, and understand the basic nutrition information and services they need to make appropriate nutrition decisions^12^. | 44.96 ± 14.97 | Female: 76 | High school degree: 15, some college or associates degree: 27, bachelor's degree: 25, graduate degree: 27 |
| McNamara, 2022 ^31^ | United States | Validation paper | College students (n=672) | A person’s ability to understand and use nutrition information to make appropriate nutrition decisions^11^. | 19.9 ± 1.8 | Female %: 62.5 | - |
| Pilut, 2022 ^32^ | United States | Randomized controlled trial | Employees at Iowa State University (n=68) | - | - | - | - |
| Rollins, 2022 ^33^ | United States | Randomized controlled trial | Black or African American expecting mother and fathers (n=80) | - |  |  |  |
| Yuen, 2018 ^15^ | United States | Systematic review | Adults | The ability to obtain, process, and understand basic nutrition information and services needed to make appropriate nutrition-related decisions^12^. |  |  |  |

**Supplementary Table 6.** Characteristics of Nutrition Literacy Studies Conducted in Taiwan, Including Study Design, Target Population, Definitions, and Sample Demographics

| **Author, Year Paper Published** | **Country** | **Study Design** | **Target**  **Population** | **Nutrition Literacy Definition** | **Age**  **(mean ± SD)** | **Gender (n)** | **Education Level (n)** |
| --- | --- | --- | --- | --- | --- | --- | --- |
| Taiwan | | | | | | | |
| Lai, 2023 ^34^ | Taiwan | Randomized controlled trial | College students at a Taiwanese university (n=98) | A type of health literacy that emphasizes obtaining, processing and understanding the nutrition information and skills needed to make appropriate nutrition-related decisions^12,15^. | 19.88 ± 1.3 | Male: 18, female: 80 | Freshman 47 (48.0), sophomore 20 (20.4), junior 18 (18.4), senior 13 (13.3) |
| Liao, 2017 ^35^ | Taiwan | Qualitative research | College Students in Taiwan (n=28 experts) | The capacity of individuals to obtain, understand, analyze, appraise, and apply nutrition-related information allowing individuals to make informed decisions, contributing to improved nutrition and health outcomes^2,12,36^. | - | - | Accredited dietitians - 85.7% |
| Liao, 2018 ^37^ | Taiwan | Validation paper | Taiwanese college students | The capacity to obtain, process, and understand nutrition information and skills needed to make appropriate nutrition decisions^2,12,28,36^. | Freshman: 237, Sophomore: 481 Junior: 330, Senior: 207 | Male: 581, female: 669 | - |

**Supplementary Table 7.** Characteristics of Nutrition Literacy Studies Conducted in other Countries including Study Design, Target Population, Definitions, and Sample Demographics.

| **Author, Year Paper Published** | **Country** | **Study Design** | **Target**  **Population** | **Nutrition Literacy Definition** | **Age**  **(mean ± SD)** | **Gender (n)** | **Education Level (n)** |
| --- | --- | --- | --- | --- | --- | --- | --- |
| Other | | | | | | | |
| Griebler, 2024 ^38^ | Austria | Validation paper | Adults in Austria (n =3,000) | Extent to which individuals are able to access, understand, appraise, and apply nutrition information needed to make appropriate everyday nutrition decisions for better health and well-being^12,23^. | aged ≥18 years | Male: 1458, female:1532, diverse gender: 3 | Compulsory education: 584, intermediate vocational/education training: 1454, A-level or higher: 956 |
| Vrinten, 2023 ^39^ | Belgium | Validation paper | Young adults (n= 505) | The ability to obtain, process, and understand nutrition information to make appropriate nutrition related decisions^12^. | 21.6 ± 2.20; 22.0 ± 2.07 | Male: 169, female: 221, non-binary: 2 | Primary: 13, secondary: 180, tertiary: 199 |
| Truman, 2020 ^23^ | Canada | Meta-review | - | A distinct subset of health literacy^12^, and a unique concept in relation to food and health literacies^11^. It focuses on a set of specialized skills that inform decisions related to nutrition, such as consuming the recommended daily servings of fruits and vegetables^12^. | - | - | - |
| Cabezas, 2023 ^40^ | Chile | Scoping review | Adults, adolescents, and school-age children | Nutrition literacy considers skills needed to obtain and understand nutrition data. | - | - | - |
| Mourouti, 2023 ^41^ | Greece | Randomized controlled trial | Hypertensive patients >40 years old (n=24) | The degree to which individuals can obtain, process, and understand basic nutrition information and nutrition services they need, to make appropriate nutritional decisions^12^. | Median (IQR) 65.5 (29.25) | Male: 4, female: 2 | Up to high school 5, Higher education 1 |
| Bhatia 2023 ^42^ | India | Systematic review | All age groups | - | - | - | - |
| Narayanan, 2022 ^43^ | India | Qualitative research | Men and women in rural India (Koraput) (1047) | The process by which individuals and communities are empowered to critically analyze their nutrition situation and engage with existing social, cultural, biological, political and environmental realities meaningfully to achieve dietary diversity and have access to safe drinking water, sanitation and adequate health services in order to optimize nutrition outcomes. | - | Male: 59, female: 49 | Non literate: 58, primary: 19, middle school: 7, secondary school: 22, graduate: 2 |
| Rochman, 2018 ^44^ | Indonesia | Quantitative research | Males and females residing in community housing in Indonesia | NI | - | Male: 14, female: 16 | - |
| Sirajuddin, 2021 ^45^ | Indonesia | Randomized controlled trial | Mothers with children aged 0 to 6 months | Maternal nutritional literacy focuses on the general ability of mothers to understand the concept and implementation of nutrition in all aspects of life, especially in a balanced diet for all age groups, particularly those prone to nutritional problems^46^. | - | - | Elementary school: 8, junior school: 28, high school: 50, college: 84 |
| Abdi, 2020 ^47^ | Iran | Mixed method sequential exploratory design | Married women aged 18-50 years visiting four healthcare centers of Sanandaj City. (n= 752) | Peoples capacity to acquire, process, and comprehend dietary information^2^. | 35.18 | - | 34.3% had diploma |
| Ramezankhan, 2023 ^48^ | Iran | Systematic review | Healthcare students | The ability to access, process, and comprehend fundamental nutrition information is known as nutrition literacy^44,49,50^. |  |  |  |
| Vettori, 2019 ^51^ | Italy | Scoping review |  | Knowledge, skills, and competence necessary for nutritional health. |  |  |  |
| Aihara, 2011^36^ | Japan | Cross-sectional study | Elderly adults >75yo living in Japan (n=678) | The degree to which people have the ability to obtain, process and understand basic diet information and the tools needed to make appropriate nutrition decisions^2,12^. | 75.83 ± 2.06 | Male:  347, female: 331 | 9 years: 208, 10 years: 283, 13 years: 180 |
| Guttersrud, 2014 ^52^ | Norway | Validation paper | Norwegian university students in nursing education. | The capacity to obtain, process and understand nutrition information and the materials needed to make appropriate decisions regarding one’s health^12^. | - | - | 96% nursing students |
| Al Tell, 2023 ^53^ | Palestine | Cross-sectional study | Young adults enrolled in bachelor’s of science degrees  (n=147) | - | 20.4 ± 4.9 | 78% female |  |
| Silva, 2023 ^54^ | Portugal | Narrative review | - | The level to which people can acquire, process, and comprehend the fundamental nutritional data and services that they need to make correct dietary decisions. ^12^ | - | - | - |
| Sousa, 2023 ^55^ | Portugal | Validation paper | Adults residing in Portugal (n = 329) | The degree to which individuals are able to obtain, process and understand nutrition and diet information, as well as access services needed to make adequate nutrition decisions ^56^ | 36.7 ± 16.1 | Male: 85, female: 239, prefers not to answer: 5 | Basic education: 38, secondary education: 86, higher education, unrelated to health area 131, higher education, health area: 53, higher education, nutrition area: 21 |
| Lobo, 2021^57^ | Spain | Validation paper | Older adults ≥ 65 and over, participating in exercise activities for seniors, living in Spain (n= 316) | Nutrition literacy refers to the knowledge, skills, and competencies necessary to maintain nutritional health^56^. | 74 ± 6 | Male: 29, female: 285 | Primary: 210, secondary/university: 100 |
| Krause, 2018 ^25^ | Switzerland | Systematic review | - | Nutrition literacy is defined as an individual’s capacity to obtain, process, and understand basic nutrition information necessary for making appropriate nutrition decisions^2,6,12,52,58,59^. |  |  |  |

1. Aihemaitijiang S, Ye C, Halimulati M, Huang X, Wang R, Zhang Z. Development and Validation of Nutrition Literacy Questionnaire for the Chinese Elderly. *Nutrients*. 2022-02-27 2022;14(5):1005. doi:10.3390/nu14051005

2. Zoellner J, Connell C, Bounds W, Crook L, Yadrick K. Nutrition literacy status and preferred nutrition communication channels among adults in the Lower Mississippi Delta. *Prev Chronic Dis*. Oct 2009;6(4):A128.

3. Kickbusch IS. Health literacy: addressing the health and education divide. *Health Promot Int*. Sep 2001;16(3):289-97. doi:10.1093/heapro/16.3.289

4. Li Z, Zhou Y, Tan Y, et al. Development and Validation of Nutrition Literacy Assessment Instrument for Chinese Lactating Women: A Preliminary Study. *Nutrients*. 2023-08-07 2023;15(15):3488. doi:10.3390/nu15153488

5. Li Z, Zhen T, Zhao Y, Zhang J. Development and assessment of a nutrition literacy scale for patients with end-stage kidney disease undergoing dialysis and its correlation with quality of life. *Renal Failure*. 2023-12-31 2023;45(1)doi:10.1080/0886022x.2022.2162417

6. Blitstein JL, Evans WD. Use of nutrition facts panels among adults who make household food purchasing decisions. *J Nutr Educ Behav*. Nov-Dec 2006;38(6):360-4. doi:10.1016/j.jneb.2006.02.009

7. Li Q, Piaseu N, Phumonsakul S, Thadakant S. Effects of a Comprehensive Dietary Intervention Program, Promoting Nutrition Literacy, Eating Behavior, Dietary Quality, and Gestational Weight Gain in Chinese Urban Women with Normal Body Mass Index during Pregnancy. *Nutrients*. 2024-01-10 2024;16(2):217. doi:10.3390/nu16020217

8. Li Q, Wang J. Effectiveness of nutrition literacy intervention on pregnancy weight and eating behavior: a randomized controlled trial. *Scientific Reports*. 2025-07-01 2025;15(1)doi:10.1038/s41598-025-07979-3

9. Liu S, Zeng H, Ma Y, Zhou Y, Wang D. Path analysis of current status and influencing factors of nutritional literacy among community-dwelling older adults. *Geriatr Nurs*. Oct 27 2023;54:264-271. doi:10.1016/j.gerinurse.2023.10.004

10. Mo G, Han S, Gao T, Sun Q, Zhang M, Liu H. Development and validation of a novel short-form nutrition literacy measurement tool for Chinese college students. *Frontiers in Public Health*. 2022-09-09 2022;10doi:10.3389/fpubh.2022.962371

11. Velardo S. The Nuances of Health Literacy, Nutrition Literacy, and Food Literacy. *Journal of Nutrition Education and Behavior*. 2015-07-01 2015;47(4):385-389.e1. doi:10.1016/j.jneb.2015.04.328

12. Silk KJ, Sherry J, Winn B, Keesecker N, Horodynski MA, Sayir A. Increasing nutrition literacy: testing the effectiveness of print, web site, and game modalities. *J Nutr Educ Behav*. Jan-Feb 2008;40(1):3-10. doi:10.1016/j.jneb.2007.08.012

13. Zhang Y, Sun Q, Zhang M, Mo G, Liu H. Nutrition Literacy Measurement Tool With Multiple Features for Chinese Adults. *Food Nutr Bull*. Jun 2022;43(2):189-200. doi:10.1177/03795721211073221

14. Taylor MK, Sullivan DK, Ellerbeck EF, Gajewski BJ, Gibbs HD. Nutrition literacy predicts adherence to healthy/unhealthy diet patterns in adults with a nutrition-related chronic condition. *Public Health Nutrition*. 2019-08-01 2019;22(12):2157-2169. doi:10.1017/s1368980019001289

15. Yuen EYN, Thomson M, Gardiner H. Measuring Nutrition and Food Literacy in Adults: A Systematic Review and Appraisal of Existing Measurement Tools. *HLRP: Health Literacy Research and Practice*. 2018-07-01 2018;2(3):e134-e160. doi:10.3928/24748307-20180625-01

16. Zhang W, Wu Y, Zhang Y, Sun Z, Wang J, Wu Y. Development and preliminary validation of the nutrition literacy scale for type 2 diabetes mellitus in China—a mixed methods study. *Frontiers in Public Health*. 2025-05-13 2025;13doi:10.3389/fpubh.2025.1569675

17. Ma RCW. Epidemiology of diabetes and diabetic complications in China. *Diabetologia*. 2018-06-01 2018;61(6):1249-1260. doi:10.1007/s00125-018-4557-7

18. Zhang W, Wang J, Wu Y, Xia Y, Sun Z, Wu Y. Development and validation of the nutrition literacy scale for Chinese gout patients. *PLOS ONE*. 2025-02-12 2025;20(2):e0318259. doi:10.1371/journal.pone.0318259

19. Nutbeam D. Health literacy as a public health goal: a challenge for contemporary health education and communication strategies into the 21st century. *Health Promotion International*. 2000-09-01 2000;15(3):259-267. doi:10.1093/heapro/15.3.259

20. Zhou Y, Lyu Y, Zhao R, et al. Development and Validation of Nutrition Literacy Assessment Instrument for Chinese Pregnant Women. *Nutrients*. 2022-07-13 2022;14(14):2863. doi:10.3390/nu14142863

21. Bedoyan J, McNamara J, Olfert MD, Byrd-Bredbenner C, Greene GW. Establishing criterion validity for the Revised Critical Nutrition Literacy Tool in U.S. college students. *J Educ Health Promot*. 2021;10:37. doi:10.4103/jehp.jehp_632_20

22. Carbone ETDRDLDN, Zoellner JMPRD. Nutrition and Health Literacy: A Systematic Review to Inform Nutrition Research and Practice. *Journal of the Academy of Nutrition and Dietetics*. 2012;112(2):254-265. doi:10.1016/j.jada.2011.08.042

23. Truman E, Bischoff M, Elliott C. Which literacy for health promotion: health, food, nutrition or media? *Health promotion international*. 2020;35(2):432-444. doi:10.1093/heapro/daz007

24. Carroll AM, Rotman Y. Nutrition Literacy Is Not Sufficient to Induce Needed Dietary Changes in Nonalcoholic Fatty Liver Disease. *Am J Gastroenterol*. Aug 1 2023;118(8):1381-1387. doi:10.14309/ajg.0000000000002182

25. Krause C, Sommerhalder K, Beer-Borst S, Abel T. Just a subtle difference? Findings from a systematic review on definitions of nutrition literacy and food literacy. *Health Promotion International*. 2016-11-01 2016:daw084. doi:10.1093/heapro/daw084

26. Gibbs H, Chapman-Novakofski K. Establishing Content Validity for the Nutrition Literacy Assessment Instrument. *Preventing Chronic Disease*. 2013-07-03 2013;10doi:10.5888/pcd10.120267

27. Gibbs H, Chapman-Novakofski K. Exploring nutrition literacy: Attention to assessment and the skills clients need. *Health*. 2012-01-01 2012;04(03):120-124. doi:10.4236/health.2012.43019

28. Gibbs HD, Ellerbeck EF, Befort C, et al. Measuring Nutrition Literacy in Breast Cancer Patients: Development of a Novel Instrument. *J Cancer Educ*. Sep 2016;31(3):493-9. doi:10.1007/s13187-015-0851-y

29. Gibbs HD, Ellerbeck EF, Gajewski B, Zhang C, Sullivan DK. The Nutrition Literacy Assessment Instrument is a Valid and Reliable Measure of Nutrition Literacy in Adults with Chronic Disease. *Journal of Nutrition Education and Behavior*. 2018;50(3):247-257.e1. doi:10.1016/j.jneb.2017.10.008

30. Marchello NJ, Daley CM, Sullivan DK, Nelson-Brantley HV, Hu J, Gibbs HD. Nutrition Literacy Tailored Interventions May Improve Diet Behaviors in Outpatient Nutrition Clinics. *Journal of Nutrition Education and Behavior*. 2021;53(12):1048-1054. doi:10.1016/j.jneb.2021.07.013

31. McNamara J, Kunicki ZJ, Neptune L, Parsons K, Byrd-Bredbenner C. Development and Validation of the Young Adult Nutrition Literacy Tool. *J Nutr Educ Behav*. Jul 2022;54(7):691-701. doi:10.1016/j.jneb.2022.01.011

32. Pilut J, Hollis J, Lanningham-Foster L, Pitchford EA, Litchfield R. Virtual Reality and Powerpoint Grocery Store Tours: Equally Effective in Improving Self-efficacy in Randomised Control Trial. *American journal of health promotion*. 2022;36(8):1346-1349. doi:10.1177/08901171221108274

33. Rollins LPMSW, Giddings TMA, Henes SPRDNLD, et al. Design and Implementation of a Nutrition and Breastfeeding Education Program for Black Expecting Mothers and Fathers. *Journal of nutrition education and behavior*. 2022;54(8):794-803. doi:10.1016/j.jneb.2022.03.011

34. Lai I-J, Chang L-C, Lee C-K, Liao L-L. Preliminary evaluation of a scenario-based nutrition literacy online programme for college students: a pilot study. *Public Health Nutrition*. 2023-12-01 2023;26(12):3190-3201. doi:10.1017/s1368980023002471

35. Liao L-L, Lai IJ. Construction of Nutrition Literacy Indicators for College Students in Taiwan: A Delphi Consensus Study. *Journal of nutrition education and behavior*. 2017;49(9):734-742.e1. doi:10.1016/j.jneb.2017.05.351

36. Aihara Y, Minai J. Barriers and catalysts of nutrition literacy among elderly Japanese people. *Health promotion international*. 02/01 2011;26:421-31. doi:10.1093/heapro/dar005

37. Liao L-L, Lai IJ, Shih S-F, Chang L-C. Development and validation of the nutrition literacy measure for Taiwanese college students. 台灣大學生營業素養測量工具之發展與驗證. *Taiwan Gong Gong Wei Sheng Za Zhi*. Oct 2018

2024-10-05 2018;37(5):582. doi:<https://doi.org/10.6288/TJPH.201810_37(5).107054>

38. Griebler R, Schütze D, Link T, Schindler K. Brief instruments for measuring nutrition literacy - the Nutrition Health Literacy Scale and the Self-Perceived Food Literacy Scale Short Form. *Nutrition Journal*. 2024-07-11 2024;23(1)doi:10.1186/s12937-024-00971-z

39. Vrinten J, Van Royen K, Pabian S, De Backer C, Matthys C. Development and validation of a short nutrition literacy scale for young adults. *Frontiers in Nutrition*. 2023-03-20 2023;10doi:10.3389/fnut.2023.1008971

40. Cabezas M-F, Nazar G. A scoping review of food and nutrition literacy programs. *Health Promotion International*. 2023;38(5)doi:10.1093/heapro/daad090

41. Mourouti N, Michou M, Lionis C, et al. An educational intervention to improve health and nutrition literacy in hypertensive patients in Greece. *Journal of Education and Health Promotion*. 2023;12(1):234-234. doi:10.4103/jehp.jehp_14_23

42. Bhatia V, Parida S, Panda M. Demographic dynamics and the changing faces of nutrition literacy in India: A tryst with the transition among communities over two decades. *Journal of International Society of Preventive and Community Dentistry*. 2023;13(4):299-306. doi:10.4103/jispcd.JISPCD_165_22

43. Narayanan R, Panda AK, Nithya DJ, Bhavani RV. Dialogue as a tool of nutrition literacy in an agricultural intervention programme in Odisha, India. *CABI agriculture and bioscience*. 2022;3(1):28-16. doi:10.1186/s43170-022-00090-x

44. Rochman C, Nasrudin D, Helsy I, Hermita N, Darmalaksana W, Nasrullah. Nutrition Literacy Program for Improving Public Wellness. *Journal of physics Conference series*. 2018;1028(1):12031. doi:10.1088/1742-6596/1028/1/012031

45. Sirajuddin, Sirajuddin S, Razak A, Ansariadi, Thaha RM, Sudargo T. The Intervention of Maternal Nutrition Literacy Has the Potential to Prevent Childhood Stunting: Randomized Control Trials. *Journal of public health research*. 2021;10(2)doi:10.4081/jphr.2021.2235

46. Mbogori TMS, Murimi MPRDLDN, Ruhul AMM. Nutrition Education Intervention: Using Train the Trainer Approach to Reach Populations With Low Literacy in Turkana, Kenya. *Journal of nutrition education and behavior*. 2015;47(4):S81-S82. doi:10.1016/j.jneb.2015.04.215

47. Abdi N, Sadeghi R, Zamani-Alavijeh F, Shojaee Zadeh D, Shahsavari S, Taghdisi MH. The Effect of Social Marketing Model on Promoting Nutrition Literacy and Healthy Dietary Behaviors of Women in Sanandaj City: A Mixed-Methods Approach. *Health Scope*. 2020-03-07 2020;9(1)doi:10.5812/jhealthscope.62753

48. Ramezankhani A, Vahidi S. Nutrition Literacy and Health Status of Medical Students: A Systematic Review. 01/03 2024;2:231-238. doi:10.58209/hehp.11.2.231

49. Buxton C, Davies A. Nutritional knowledge levels of nursing students in a tertiary institution: Lessons for curriculum planning. *Nurse education in practice*. 2013;13(5):355-360. doi:10.1016/j.nepr.2012.09.014

50. Hemati M, Toori MA, Shams M, Behroozpour A, Rezaei A. Measuring Nutritional Literacy in Elementary School Teachers in Yasuj: A Cross-Sectional Study. *Armaghān-i dānish*. 2018;23(1):124-133.

51. Vettori V, Lorini C, Milani C, Bonaccorsi G. Towards the Implementation of a Conceptual Framework of Food and Nutrition Literacy: Providing Healthy Eating for the Population. *Int J Environ Res Public Health*. Dec 11 2019;16(24)doi:10.3390/ijerph16245041

52. Guttersrud Ø, Dalane JØ, Pettersen S. Improving measurement in nutrition literacy research using Rasch modelling: examining construct validity of stage-specific ‘critical nutrition literacy’ scales. *Public Health Nutrition*. 2014-04-01 2014;17(4):877-883. doi:10.1017/s1368980013000530

53. Al Tell M, Natour N, Alshawish E, Badrasawi M. The relationship between nutrition literacy and nutrition information seeking attitudes and healthy eating patterns among a group of palestinians. *BMC public health*. 2023;23(1):165-8. doi:10.1186/s12889-023-15121-z

54. Silva P, Araújo R, Lopes F, Ray S. Nutrition and Food Literacy: Framing the Challenges to Health Communication. *Nutrients*. Nov 7 2023;15(22)doi:10.3390/nu15224708

55. Sousa S, Albuquerque G, Severo M, et al. Development and validation of a photograph-based instrument to assess nutrition literacy: The NUTLY project. *Appetite*. Jul 1 2024;198:107377. doi:10.1016/j.appet.2024.107377

56. Vettori V, Lorini C, Milani C, Bonaccorsi G. Towards the Implementation of a Conceptual Framework of Food and Nutrition Literacy: Providing Healthy Eating for the Population. *International journal of environmental research and public health*. 2019;16(24):5041. doi:10.3390/ijerph16245041

57. Lobo E, Tamayo M, Sanclemente T. Nutrition Literacy and Healthy Diet: Findings from the Validation of a Short Seniors-Oriented Screening Tool, the Spanish Myths-NL. *International Journal of Environmental Research and Public Health*. 2021-11-18 2021;18(22):12107. doi:10.3390/ijerph182212107

58. Watson WL, Chapman K, King L, et al. How well do Australian shoppers understand energy terms on food labels? *Public health nutrition*. 2013;16(3):409-417. doi:10.1017/S1368980012000900

59. Neuhauser LD, Rothschild RMPA, Rodríguez FMMPH. MyPyramid.gov: Assessment of Literacy, Cultural and Linguistic Factors in the USDA Food Pyramid Web Site. *Journal of nutrition education and behavior*. 2007;39(4):219-225. doi:10.1016/j.jneb.2007.03.005
