# Supplementary material for: A Conceptual Nutrition Literacy Framework for Adults in the United States: A Scoping Review and Thematic Analysis
Source: Adv Nutr. 2026 Jun 18;17(8):100686. doi: 10.1016/j.advnut.2026.100686 (PMC13382296; doi:10.1016/j.advnut.2026.100686)
Supplement: Multimedia component 2 [file mmc2.pdf]

A Conceptual Nutrition Literacy Framework for Adults in the United States: A Scoping Review and Thematic Analysis. Aubree L. Hawley et al.

UNIVERSITY OF ARKANSAS

Introduction

Background and Overall Goal: Nutrition literacy assessment and intervention requires a comprehensive, standardized framework to effectively guide practice and research. Currently, there is no comprehensive framework that assesses all key components of nutrition literacy that is validated in the United States. University of Arkansas researchers are attempting to create such a framework.

Audience The nutrition literacy framework will be used with the general population in the U.S to assist with program development, assessment tool development, and to advance the field of nutrition literacy.

You will be asked to evaluate the overall framework, domains, levels, and level examples. You will also have the opportunity to provide open-ended feedback to inform modifications.

Depending on the amount of feedback provided, we estimate that up to 1-hours may be required to complete this task. You are able to stop at anytime and your progress will be saved.

Basic Info

Name (first and last)

Email Address

[ ]

Which of the following activities have you ever been employed to conduct? Select all that apply

- Nutrition research
- Public health research, education, and/or teaching
- Dietary/nutrition assessment
- Nutrition education, teaching, and/or counseling
- [ ] Other

About how many years of work experience do you have in the nutrition, physical activity/exercise, public health, and/or education space?

- Less than 1 year
- 1 to 4 years
- 5 to 9 years
- 10-20 years
- Over 20 years

Are you a Registered Dietitian?

- Yes
- No

What is the highest degree you have obtained?

- ◯ Bachelor's Degree (B.A., B.S.)
- ◯ Master's Degree (M.A., M.S., M.E.d.)
- ◯ Doctoral Degree (Ph.D., Ed.D.)
- ◯ Postdoctoral or Higher

Framework Overview

Instructions: We are seeking your overall feedback on the nutrition literacy framework. Please rate the following statements based on your agreement using the five-point Likert scale below: 1 = Strongly Disagree 2 = Disagree 3 = Neutral 4 = Agree 5 = Strongly Agree.

A Nutrition Literacy Framework

Nutrition Literacy (NL) Definition: **Nutrition literacy (NL) refers to the degree to which an individual has the capacity to obtain, process, and understand nutrition information and services required for making appropriate nutrition decisions. Velardo, S. (2015)**

Framework	Domain	Definition	Levels	Dimension (Cognition vs skill)	Definition	Example 1	Example 2
Nutrition Literacy	Functional	The ability to understand, obtain, and apply nutritional information or nutrition services.	Knowledge	Cognition	Basic nutrition knowledge.	**Knowledge Example A**: Identify portion sizes, food groups classification, recognize nutrient-dense foods, identify foods with nutrients emphasized by the DGA to include (e.g. fiber) or limit (e.g. added sugar).	**Knowledge Example B:** Awareness of MyPlate and/or Dietary Guidelines for Americans recommendations.
			Understand (Krause, 2018)	Cognition	The ability to read and comprehend nutrition information and dietary advice	**Understand Example A:** Read and understand the nutritional information on food packages or food labels.	**Understand Example B:** The ability to read about healthy fats and foods containing healthy fats and subsequently identify items that contain healthy fats.
			Obtaining	Skill	The ability to search for, find, and obtain nutrition information or services	**Obtain Example A:** The ability to find trustworthy and reliable nutrition information.	**Obtain Example B:** The ability to find a health professional to talk to about personal nutrition information.
			Applying	Skill	The ability to apply nutrition information or nutrition services to achieve a healthy diet (short term)	**Apply Example A**: I can use a nutrition facts panel to identify products with added sugar.	**Apply Example B:** I can use a nutrition facts panel to pick healthier foods.
	Interactive	Advanced cognitive and communication skills that enable individuals to effectively interact with, communicate about, and apply relevant nutrition information to support changing health and wellness needs.	Advanced Cognitive and Application Skills (Guttersrud, 2014)(Vrinten, 2023)	Cognition and Skill	The ability to apply food and nutrition information in-store, online, and at home to consume a dietary pattern that aligns with the Dietary Guidelines for Americans (DGA) or recommendation from health professionals for personal health or health of others.	**Advanced Cognitive and Application Skills Example A:** The ability to know saturated fat increases heart disease risk, the ability to choose low-saturated fat products, and the ability to plan a meal low in saturated fat.	**Advanced Cognitive and Application Skills Example B:** The ability to apply nutrition knowledge to plan meals that meet both the Dietary Guidelines for Americans and healthcare provider recommendations.
			Motivation	Cognition	The intrinsic drive and sustained commitment to consistently acquire, implement and act upon credible nutrition information to establish and maintain healthy dietary patterns aligned with one's goals and circumstances.	**Motivation Example A:** The drive to seek out nutrition information for the purpose of improving one's nutritional status and behavior.	**Motivation Example B:** The drive to take initiative to discuss healthy eating with a health professional such as a medical doctor or registered dietitian.
			Communicative (McNamara, 2021)	Skill	(2) The ability to listen, speak, negotiate, and engage in-person and in a digital environment to support individual nutrition needs and apply relevant nutrition information.	**Communicative Example A:** The ability to effectively communicate nutrition concerns and ask relevant questions during healthcare provider consultations.	**Communication Example B:** The ability to communicate reliable nutrition information with family and friends, distinguishing between credible dietary advice and popular misconceptions when discussing healthy eating strategies.
	Critical	The ability to critically analyze, assess, and adapt nutrition information, advocate for personal and community nutrition needs, and translate knowledge into action while addressing barriers to healthy eating practices.	Appraisal (Guttersrud, 2014)(Vrinten, 2023)	Skill	The critical evaluation and judgment of digital and non-digital nutrition information, marketing cues, and dietary advice in the context of personal and community needs.	**Appraisal Example A:** The ability to tell what nutrition information is reliable and what is not.	**Appraisal Example B:** The ability to reject untrue nutrition advice from family or friends.
			Advocacy (Guttersrud, 2014)(Vrinten, 2023)	Skill	The capacity and willingness to take action in promoting equitable access to healthy eating by addressing barriers and influencing policies and practices related to nutrition.	**Advocacy Example A:** Concerned that the price of food that is considered to be healthy may get too high	**Advocacy Example B:** Knowing the importance of engaging in community initiatives that advocate for equitable access to healthy food and nutrition resources for all people.
			Translational (Stanley, 2022) (Vrinten, 2023) (Guttersrud, 2014)	Skill	(2) The ability to adapt or transfer healthy nutrition behaviors to new technologies, contexts, and situations and to advocate for one's nutrition and dietary needs.	**Translational Example A:** The ability to apply nutrition knowledge to implement strategies that encourage healthy eating habits and promote access to nutritious foods in schools, workplaces, or community settings.	**Translational Example B:** The ability to apply nutritional knowledge to guide and support healthy eating choices for family members and friends, fostering long-term habits that contribute to overall well-being.

	Strongly Disagree	Disagree	Neutral	Agree	Strongly Agree
The framework provides a logical and coherent structure for understanding nutrition literacy.	○	○	○	○	○
The framework provides enough information to understand how it can be applied to research and real-world settings such as assessment tool, program, and intervention development.	○	○	○	○	○

Nutrition Literacy Framework Overall: Please share any feedback or suggestions that could help improve the final version of the proposed framework.

Domain Evaluation

Instructions: We are seeking your feedback on each key domain in the nutrition literacy framework. Please rate the following statements based on your agreement with the definition, using the five-point Likert scale below: 1 = Strongly Disagree 2 = Disagree 3 = Neutral 4 = Agree 5 = Strongly Agree Your responses will help us assess the comprehensiveness of the framework's definitions for each domain of nutrition literacy.

Functional Nutrition Literacy: The ability to understand, obtain, and apply nutritional information or nutrition services.

	Strongly Disagree	Disagree	Neutral	Agree	Strongly Agree
The functional literacy definition is easy to understand.	○	○	○	○	○
The functional literacy definition is straightforward and free from ambiguity.	○	○	○	○	○
The functional literacy definition represents the key components of functional nutrition literacy.	○	○	○	○	○

Interactive Nutrition Literacy: Advanced cognitive and communication skills that enable individuals to effectively interact with, communicate about, and apply relevant nutrition information to support changing health and wellness needs.

	Strongly Disagree	Disagree	Neutral	Agree	Strongly Agree
The interactive literacy definition is easy to understand.	○	○	○	○	○
The interactive literacy definition is straightforward and free from ambiguity.	○	○	○	○	○
The interactive literacy definition represents the key components of interactive nutrition literacy.	○	○	○	○	○

Critical nutrition literacy: The ability to critically analyze, assess, and adapt nutrition information, advocate for personal and community nutrition needs, and translate knowledge into action while addressing barriers to healthy eating practices.

	Strongly Disagree	Disagree	Neutral	Agree	Strongly Agree
The critical literacy definition is easy to understand.	○	○	○	○	○
The critical literacy definition is straightforward and free from ambiguity.	○	○	○	○	○
The critical literacy definition represents the key components of critical nutrition literacy.	○	○	○	○	○

Nutrition Literacy Domains Overall: Please include any overall

feedback below.

Functional Levels

Functional Literacy Instructions: Functional nutrition literacy contains four levels: knowledge, understanding, obtaining, and applying nutritional information. Please rate the following statements based on your agreement with the level definition, using the five-point Likert scale below: 1 = Strongly Disagree 2 = Disagree 3 = Neutral 4 = Agree 5 = Strongly Agree

Nutrition Functional Literacy

Domain	Definition	Levels	Definition	
Functional	The ability to understand, obtain, and apply nutritional information or nutrition services.	Knowledge	Basic nutrition knowledge.	[illegible]
		Understand (Krause, 2018)	The ability to read and comprehend nutrition information and dietary advice	[illegible]
		Obtaining	The ability to search for, find, and obtain nutrition information or services	[illegible]
		Applying	The ability to apply nutrition information or nutrition services to achieve a healthy diet (short term)	[illegible]

	Strongly Disagree	Disagree	Neutral	Agree	Strongly Agree
The knowledge level definition is straightforward and free from ambiguity.	○	○	○	○	○
The understand level definition is straightforward and free from ambiguity.	○	○	○	○	○
The obtain level definition is straightforward and free from ambiguity.	○	○	○	○	○
The apply level definition is straightforward and free from ambiguity.	○	○	○	○	○

Instructions: We are seeking your feedback on level examples in the nutrition literacy framework. Please rate the following statements based on your agreement with the statements regarding the level examples, using the five-point Likert scale below: 1 = Strongly Disagree 2 = Disagree 3 = Neutral 4 = Agree 5 = Strongly Agree.

Functional Nutrition Literacy: The ability to understand, obtain, and apply nutritional information or nutrition services.

Example 1	Example 2
**Knowledge Example A**: Identify portion sizes, food groups classification, recognize nutrient-dense foods, identify foods with nutrients emphasized by the DGA to include (e.g. fiber) or limit (e.g. added sugar).<br>**Understand Example A:** Read and understand the nutritional information on food packages or food labels.<br>**Obtain Example A:** The ability to find trustworthy and reliable nutrition information.<br>**Apply Example A**: I can use a nutrition facts panel to identify products with added sugar.	**Knowledge Example B:** Awareness of MyPlate and/or Dietary Guidelines for Americans recommendations.<br>**Understand Example B:** The ability to read about healthy fats and foods containing healthy fats and subsequently identify items that contain healthy fats.<br>**Obtain Example B:** The ability to find a health professional to talk to about personal nutrition information.<br>**Apply Example B:** I can use a nutrition facts panel to pick healthier foods.

	Strongly Disagree	Disagree	Neutral	Agree	Strongly Agree
The Knowledge Level examples are easy to understand.	○	○	○	○	○
The Knowledge Level examples align well with the level definition provided.	○	○	○	○	○
The Understand Level examples are easy to understand.	○	○	○	○	○
The Understand Level examples align well with the level definition provided.	○	○	○	○	○
The Obtain Level examples are easy to understand	○	○	○	○	○
The Obtain Level examples align well with the level definition provided	○	○	○	○	○
The Apply Level examples are easy to understand.	○	○	○	○	○
The Apply Level examples align well with the level definition provided	○	○	○	○	○

Functional Domain and Level Examples Overall: Please include any overall feedback below. If the functional literacy domain levels needs to be adjusted, please state so here.

Interactive Levels

Interactive	Advanced cognitive and communication skills that enable individuals to effectively interact with, communicate about, and apply relevant nutrition information to support changing health and wellness needs.	Advanced Cognitive and Application Skills (Guttersrud, 2014)(Vrinten, 2023)	The ability to apply food and nutrition information in-store, online, and at home to consume a dietary pattern that aligns with the Dietary Guidelines for Americans (DGA) or recommendation from health professionals for personal health or health of others.
		Motivation	The intrinsic drive and sustained commitment to consistently acquire, implement and act upon credible nutrition information to establish and maintain healthy dietary patterns aligned with one's goals and circumstances.
		Communicative (McNamara, 2021)	(2) The ability to listen, speak, negotiate, and engage in-person and in a digital environment to support individual nutrition needs and apply relevant nutrition information.

Interactive Literacy Instructions: Interactive nutrition literacy contains three levels: Advanced cognitive and application skills, Motivation, and Communicative. Please rate the following statements based on your agreement with the level definition, using the five-point Likert scale below: 1 = Strongly Disagree 2 = Disagree 3 = Neutral 4 = Agree 5 = Strongly Agree

	Strongly Disagree	Disagree	Neutral	Agree	Strongly Agree
The Advanced Cognitive and Application Skill level definition is straightforward and free from ambiguity.	○	○	○	○	○
The Motivation level definition is straightforward and free from ambiguity.	○	○	○	○	○
The Communicative level definition is straightforward and free from ambiguity.	○	○	○	○	○

Instructions: We are seeking your feedback on level examples in the nutrition literacy framework. Please rate the following statements based on your agreement with the statements regarding the level examples, using the five-point Likert scale below: 1 = Strongly Disagree 2 = Disagree 3 = Neutral 4 = Agree 5 = Strongly Agree.

Interactive Nutrition Literacy Definition: Advanced cognitive and communication skills that enable individuals to effectively interact with, communicate about, and apply relevant nutrition information to support changing health and wellness needs.

Interactive Nutrition Literacy Examples:

**Advanced Cognitive and Application Skills Example A:** The ability to know saturated fat increases heart disease risk, the ability to choose low-saturated fat products, and the ability to plan a meal low in saturated fat.	**Advanced Cognitive and Application Skills Example B:** The ability to apply nutrition knowledge to plan meals that meet both the Dietary Guidelines for Americans and healthcare provider recommendations.
**Motivation Example A:** The drive to seek out nutrition information for the purpose of improving one's nutritional status and behavior.	**Motivation Example B:** The drive to take initiative to discuss healthy eating with a health professional such as a medical doctor or registered dietitian.
**Communicative Example A:** The ability to effectively communicate nutrition concerns and ask relevant questions during healthcare provider consultations.	**Communication Example B:** The ability to communicate reliable nutrition information with family and friends, distinguishing between credible dietary advice and popular misconceptions when discussing healthy eating strategies.

	Strongly Disagree	Disagree	Neutral	Agree	Strongly Agree
Advanced Cognitive and Application Skills examples are easy to understand	○	○	○	○	○
Advanced Cognitive and Application Skills examples align well with the level definition provided.	○	○	○	○	○
Motivation examples are easy to understand.	○	○	○	○	○
Motivation examples align well with the level definition provided.	○	○	○	○	○
Communicative examples are easy to understand.	○	○	○	○	○
Communicative examples align well	○	○	○	○	○

with the level definition provided

Interactive Literacy Domain and Level Examples Overall: Please include any overall feedback below. If the interactive literacy level definition needs to be adjusted, please state so here.

Critical Levels

Critical Literacy Domain Instructions

The following framework items pertain to critical nutrition literacy across three levels: appraisal, advocacy, and translation. These questions evaluate higher-order thinking skills in nutrition decision-making and advocacy.

Please rate the following statements based on your agreement statements regarding the level definitions, using the five-point Likert scale below: 1 = Strongly Disagree 2 = Disagree 3 = Neutral 4 = Agree 5 = Strongly Agree

Critical	The ability to critically analyze, assess, and adapt nutrition information, advocate for personal and community nutrition needs, and translate knowledge into action while addressing barriers to healthy eating practices.	Appraisal (Guttersrud, 2014)(	The critical evaluation and judgment of digital and non-digital nutrition information, marketing cues, and dietary advice in the context of personal and community needs.
		Advocacy (Guttersrud, 2014)(	The capacity and willingness to take action in promoting equitable access to healthy eating by addressing barriers and influencing policies and practices related to nutrition.
		Translational (Stanley, 2022) (Vrinten, 2023) (Guttersrud, 2014)	(2) The ability to adapt or transfer healthy nutrition behaviors to new technologies, contexts, and situations and to advocate for one's nutrition and dietary needs.

	Strongly Disagree	Disagree	Neutral	Agree	Strongly Agree
The appraisal level definition is straightforward and free from ambiguity.	○	○	○	○	○
The advocacy level definition is straightforward and free from ambiguity.	○	○	○	○	○
The translational level definition is straightforward and free from ambiguity.	○	○	○	○	○

Instructions: We are seeking your feedback on level examples in the nutrition literacy framework.

Critical Literacy Domain Definition: The ability to critically analyze, assess, and adapt nutrition information, advocate for personal and community nutrition needs, and translate knowledge into action while addressing barriers to healthy eating practices.

Please rate the following statements based on your agreement with the statements regarding the level examples, using the five-point Likert scale below: 1 = Strongly Disagree 2 = Disagree 3 = Neutral 4 = Agree 5 = Strongly Agree.

**Appraisal Example A:** The ability to tell what nutrition information is reliable and what is not.	**Appraisal Example B:** The ability to reject untrue nutrition advice from family or friends.
**Advocacy Example A:** Concerned that the price of food that is considered to be healthy may get too high	**Advocacy Example B:** Knowing the importance of engaging in community initiatives that advocate for equitable access to healthy food and nutrition resources for all people.
**Translational Example A:** The ability to apply nutrition knowledge to implement strategies that encourage healthy eating habits and promote access to nutritious foods in schools, workplaces, or community settings.	**Translational Example B:** The ability to apply nutritional knowledge to guide and support healthy eating choices for family members and friends, fostering long-term habits that contribute to overall well-being.

	Strongly Disagree	Disagree	Neutral	Agree	Strongly Agree
Appraisal examples are easy to understand	○	○	○	○	○
Appraisal examples align well with the level definition provided.	○	○	○	○	○
Advocacy examples are easy to understand	○	○	○	○	○
Advocacy examples align well with the level definition provided	○	○	○	○	○
Translational examples are easy to understand	○	○	○	○	○
Translational examples align well with the level definition provided	○	○	○	○	○

Critical Literacy Domain and Level Examples Overall: Please include any overall feedback below. If the critical literacy domain levels needs to be adjusted, please state so here.

Powered by Qualtrics
